# A Novel Online Position Estimation Method and Movement Sonification System: The Soniccup

**DOI:** 10.3390/s24196279

**Published:** 2024-09-28

**Authors:** Thomas H. Nown, Madeleine A. Grealy, Ivan Andonovic, Andrew Kerr, Christos Tachtatzis

**Affiliations:** 1 Department of Biomedical Engineering, University of Strathclyde, Glasgow G4 0UW, UK; 2 Department of Psychological Science and Health, University of Strathclyde, Glasgow G1 1QE, UK; 3 Department of Electronic and Electrical Engineering, University of Strathclyde, Glasgow G1 1XW, UKchristos.tachtatzis@strath.ac.uk (C.T.)

**Keywords:** inertial measurement unit, dead reckoning, Kalman filter, zero-velocity updates, movement sonification, upper limb motion

## Abstract

Existing methods to obtain position from inertial sensors typically use a combination of multiple sensors and orientation modeling; thus, obtaining position from a single inertial sensor is highly desirable given the decreased setup time and reduced complexity. The dead reckoning method is commonly chosen to obtain position from acceleration; however, when applied to upper limb tracking, the accuracy of position estimates are questionable, which limits feasibility. A new method of obtaining position estimates through the use of zero velocity updates is reported, using a commercial IMU, a push-to-make momentary switch, and a 3D printed object to house the sensors. The generated position estimates can subsequently be converted into sound through sonification to provide audio feedback on reaching movements for rehabilitation applications. An evaluation of the performance of the generated position estimates from a system labeled ‘Soniccup’ is presented through a comparison with the outputs from a Vicon Nexus system. The results indicate that for reaching movements below one second in duration, the Soniccup produces positional estimates with high similarity to the same movements captured through the Vicon system, corresponding to comparable audio output from the two systems. However, future work to improve the performance of longer-duration movements and reduce the system latency to produce real-time audio feedback is required to improve the acceptability of the system.

## 1. Introduction

The World Health Organization predicts that 86 million people will have an estimated 18 years of life lived with disability as a consequence of stroke [1]. Upper limb impairment is common for survivors of stroke [2], and rehabilitation is highly sought after [3,4]. Given this need, rehabilitation researchers are investigating a variety of tools to improve functional competency in performing daily living activities and enhance survivor competency to live independently. One technique reported to elicit functional improvements for stroke survivors is movement sonification, a technology that translates kinematic data into an auditory output relayed to the movement performer as feedback [5,6,7], typically achieved through a linear parameter mapping strategy. The technology grants concurrent audio feedback of the movement performed, without conflicting with the visual attention required to perform actions. In theory, this provides an additional sensory channel for the movement performer to enhance sensorimotor learning [8], which could improve motor skill learning, especially when applied in ‘real time’ [9]. However, the relatively small sample sizes in reported studies have limited the evidence of impact. Questions remain on the optimum methodology to derive the maximum benefit of movement sonification as an upper limb rehabilitation intervention, and more extensive data are required to corroborate that the intervention delivers effective improvements post intervention. As such, the need for large-scale, randomized, and managed trials remains [10].

**Figure 1:**
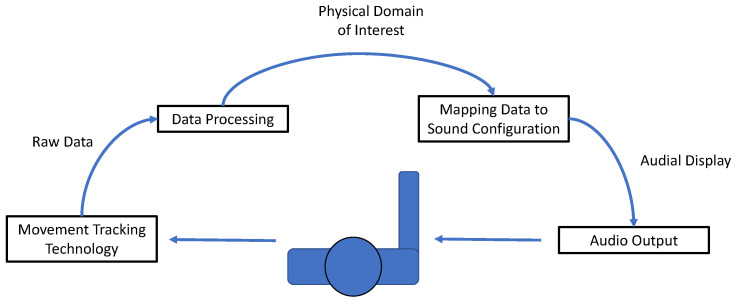
Rudimentary example of a movement sonification system. The stages of the system occur sequentially, starting with the capture of performed movement, the extraction and processing of data, translation into the auditory domain, and the playback of audio as a mode of feedback to the movement performer.

A movement sonification system (Figure 1) that is readily deployable and minimally invasive and provides data consistently is thus needed to execute more systematic studies. Presently, commercial movement sonification systems do not exist; therefore, the development of a system that facilitates large-scale studies would be a major step toward evaluating the effectiveness of movement sonification as a rehabilitation intervention.

A key component in movement sonification is the motion capture system used to acquire movement kinematic data. Reviews detailing existing real-time movement sonification systems [11] and systems in sport applications [12] have identified a number of motion capture systems available for purchase, each with different competencies and limitations. The system requirement criteria limit the suitability of many solutions; however, one is through the use of micro-electro-mechanical system-type (MEMS) inertial sensors, which have achieved ubiquitous use in smart phones and a number of other commercialized technologies characterized by human–computer interaction. The commonality of these sensors in a competitive market has resulted in the high availability of a low-cost motion capture technology. The main challenge with these sensors is susceptibility to environmental conditions [13], which introduce errors as a function of the inertial sensor type. To reduce these errors, inertial sensors are packaged and integrated with additional sensors to create an Inertial Measurement Unit (IMU). Commercial IMUs typically provide gravity-free acceleration and orientation as outputs.

IMUs have been used as the primary motion capture system in a number of movement sonification projects [11] including upper limb rehabilitation through the use of multiple IMUs to indirectly obtain upper limb joint angles using knowledge of segment lengths to, therefore, obtain hand/wrist position [7,14]. Positional tracking in movement sonification permits concurrent audiovisual feedback of movement, enabling the integration of augmented sensorimotor feedback [9]. However, the number of sensors required consequently increases the acquisition cost and setup time, scaling as a function of the number of sensors. The use of a single IMU sensor would be advantageous with respect to lowering usability barriers.

The well-reported dead reckoning method can be used to obtain position from acceleration from a single IMU [15]. Dead reckoning uses a combination of the last known position and movement kinematic data (such as acceleration) to estimate the current position. However, due to the errors associated with IMU motion capture [16], double-integration gravity-free acceleration values from the IMU to obtain position estimates can lead to consequential errors that can be orders of magnitude greater than the ‘true’ position [17], referred to as ‘integration drift’. As such, methods to remove errors associated with IMU sensors and/or mitigate integration drift are required to improve position estimates, and the creation of such method remains a challenge at present.

A method using a combination of filters and the ‘zero velocity updates (ZUPT)’ technique to condition acceleration data and improve position estimates is presented here. Kalman filters [18], used in this method, are recursive estimation algorithms that operate in a prediction/correction loop. The correction phase of the cycle receives external measurements and adjusts the predicted output based on the quality of the measurement. If the measurements are of lower quality i.e., greatly affected by noise, then new predictions will be influenced more significantly by previous predictions and less by the current measurement, with the opposite being true as well. ZUPT [19] utilizes known periods of non-movement to remove errors. In the context of obtaining position, velocity estimates obtained from integrating acceleration measurements contain associated errors. Through identifying regular periods of non-movement, these velocity estimates can be conditioned to remove errors prior to subsequent integration into position estimates.

Regular non-movement markers have been used in gait tracking applications [15] and for upper limb tracking. Comotti et al. [20] created an extended arm swing application by applying a 2 Hz low-pass filter to the measured acceleration magnitude and using a value threshold to identify movement and stationary periods. Bai et al. [21] created an application for use with the ‘Nine Hole Peg Test’ [22], a standardized clinical assessment of finger dexterity, through the use of different value thresholds determined by short-time energy calculations, and a separate threshold to identify values crossing zero. Results from these projects indicated that the use of ZUPT has merit in improving position estimates through the dead reckoning method; however, the solutions presented are tailored to specific movement actions. Conventional methods of upper limb rehabilitation use a wider range of movements as part of therapy, and often include the repetitive reaching and grasping of objects as part of the protocol. To enable this type of movement practice, an interactive handheld object was created to permit online audio feedback based on the reaching displacement achieved. The technology, referred to as ‘Soniccup’ in this paper, comprises a handheld object and an associated position estimation algorithm. Results are presented for an evaluation of the system output (position estimates), using a gold-standard motion capture system as truth data.

The formulation of generating position estimates from gravity-free linear acceleration data obtained from a single NGIMU device is at the core of the implementation. The strategy applied to obtain position estimates uses a combination of Kalman filters, ZUPT, and a ‘push-to-make’ momentary switch, integrated into a 3D printed housing to create an interactive handheld tool. Soniccup, an online movement sonification system that creates audio feedback through translating hand position estimates into audio pitch, was developed for repetitive forward reaching movements along a single axis, with the intention that movement performers lift and place object at each extremity of their movements. Although the system description focuses on a single axis of movement, relatively minor adjustments to the system can treat different types of reciprocal movements and produce audio feedback based on multiple axes.

This paper focuses on the design, implementation, and evaluation of the position estimation algorithm associated with the system. As the Soniccup is intended to provide meaningful audio feedback on a reaching movement, the profile of the captured data, as opposed to the absolute value of the data, is the metric of interest. The manuscript is organized as follows; Section 2 describes the design of the system; Section 3 details a protocol to evaluate the validity of the position estimates obtained through the Soniccup, for the purposes of movement sonification; Section 4 presents the results; and Section 5 carries out a critical assessment of the findings of this study, along with a description of future work.

## 2. Materials and Methods

### 2.1. Hardware

The system utilizes the motion capture capabilities of a Next-Generation Inertial Measurement Unit (NGIMU) from x-io Technologies Limited (Bristol, UK [23]), comprising a 3D accelerometer, 3D gyroscope, magnetometer, and sensors to monitor environmental conditions, all powered through a rechargeable 1000 mAh battery.

The NGIMU also contains an analogue input interface enabling the integration of a non-latching switch as part of the system design. The switch chosen has a low operating force permitting switch activation by object weight alone, and was connected via a stripboard that links the 3.3 V supply from the NGIMU to a voltage divider. The result is a voltage signal that feeds low/high values based on the open/closed state of the switch. The device transmits gravity-free acceleration data in the Earth reference frame and voltage data corresponding to the switch state through Wi-Fi to a host PC at 100 Hz for subsequent data processing. Gravity-free acceleration was accessible via an Attitude and Heading Reference Systems algorithm [24] available through the NGIMU sensor.

**Figure 2:**
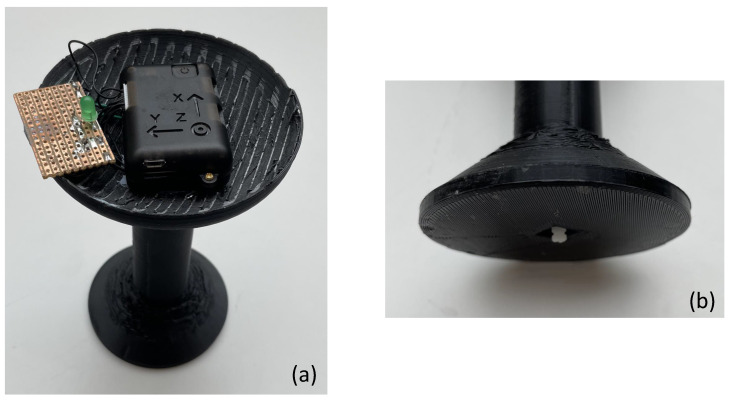
Images showing the hardware components used in the Soniccup system. Image (**a**) shows the Soniccup placed on the table. An NGIMU sensor plus stripboard are attached to the top of the 3D printed object. The stripboard contains analogue electronic components used to connect a push-to-make switch to the NGIMU. Image (**b**) shows the protruded segment of the push-to-make switch at the bottom of the Soniccup.

Figure 2 shows the constructed Soniccup, which includes the NGIMU device, stripboard with integrated voltage divider, switch, light-emitting diode for debugging purposes, and a 3D printed object designed to house sensors and provide a stable platform for placement. The integrated switch (shown in Figure 2b) was positioned flush to a flat surface when fully compressed, and the connecting wires were fed through the hollow stem of the 3D printed housing to the stripboard.

### 2.2. Position Estimation

**Figure 3:**
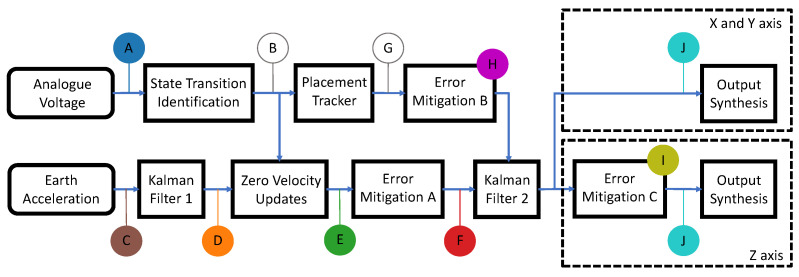
Block diagram showing the signal conditioning steps of the sonification stage, starting from analogue input and Earth acceleration.

Data streams corresponding to the acceleration and switch state were wirelessly transmitted to a host PC at 100 Hz for processing. Figure 3 shows an overview of the end-to-end processing to convert these two data streams into estimated position. The estimated position data are subsequently converted to Musical Instrument Digital Interface (MIDI) notes to produce audio feedback.

The generation of the ZUPT trigger signal and the conversion process of acceleration to position is described further to detail the methodology used to estimate position. Throughout this description, the focus is the primary direction of travel along the sagittal axis (labeled as the *X*-axis), unless stated otherwise. A positive change in the *X*-axis represents an arm extension movement, whereas a negative change represents an arm retraction movement.

#### 2.2.1. State Transition Identification

**Figure 4:**
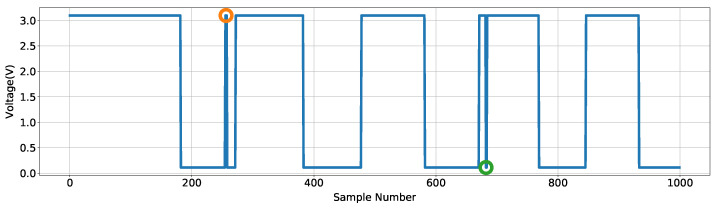
Figure depicting the recording of mechanical bouncing. Two events are shown with orange and green circles, corresponding to a momentary placement and momentary lift of the Soniccup, respectively.

The voltage data stream, represented as point ‘A’ in Figure 3, is processed to identify the placement or lift of the Soniccup from a surface. Figure 4 shows the data stream ranging between 0 and 3.3 V; evident are the momentary fluctuations of the voltage near the placement and lift events, highlighted by orange and green markers. The orange marker represents a ‘low-to-high’ transition, indicating that the switch had been momentarily compressed, while the green marker represents a ‘high-to-low’ transition, indicating the momentary release of the switch.

Fluctuations typically occur due to imperfect placement or lift, a form of ‘biomechanical bounce’, and are separate artifacts from a purposeful placement/lift. As the accurate identification of placements and lifts is a necessity for ZUPT implementation, the detection and removal of these events are mandatory. Filtering is the engineering route to removing these momentary fluctuations, triggered through actuating a state change only if the voltage signal persists for *n* consecutive samples. The process result is referred to below as the ‘switch state signal’. Additionally, as the lift of the Soniccup corresponds to movement initiation, the first switch state sample would occur at the first integrated acceleration sample (for each movement), if not before, for the described method to be effective. Failure to achieve this would result in the zeroing of movement data, leading to poor position estimates. Therefore, to conserve data at the beginning of motion, the value of *n* in this filter also determines the sample delay applied to the integrated acceleration signal, as a consequence of this process. A small value for *n* runs the risk of momentary changes remaining, while a high *n* will lead to increased lag between the movement and audio generation, which is also undesirable. A trade-off evaluation determined through trial-and-error and chosen based on the smallest value that consistently filtered the momentary fluctuations, indicated that an *n* of eight was optimum with a concomitant delay of 80 ms. Details of all temporal delays in this methodology description are shown in Appendix B. The subsequent stage—point ‘B’ on Figure 3—inputs the filtered switch state signal to ZUPT to generate the estimated velocity data.

#### 2.2.2. Acceleration to Position

**Figure 5:**
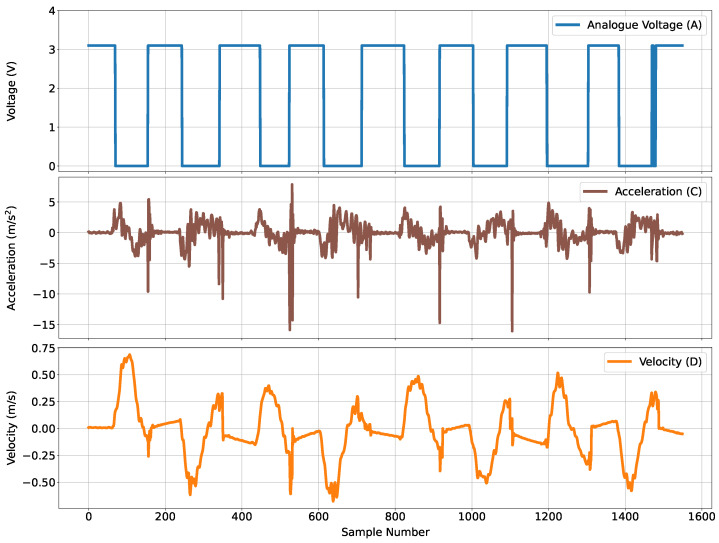
Figure showing associated data prior to and with the first designed Kalman filter: (**top**) plot of analogue voltage obtained through NGIMU, (**middle**) plot of raw data values corresponding to acceleration in Earth reference frame obtained through NGIMU sensor, (**bottom**) output estimated velocity from first Kalman filter.

The acceleration data from the sensor—‘C’ on Figure 3—is fed through a linear Kalman filter to output an estimated velocity; the filter integrates acceleration data through a white noise filter. Figure 5 shows input data obtained through the NGIMU, prior to and after the application of the Kalman filter. The top plot shows the switch voltage measurements obtained from the NGIMU; the middle plot shows the raw Earth acceleration labeled as ‘Acceleration (C)’; the bottom plot shows the output of the Kalman filter, i.e., the estimated velocity, labeled as ‘Velocity (D)’. Clearly evident is that the estimated velocity contains errors that significantly impact position estimates upon integration.

**Figure 6:**
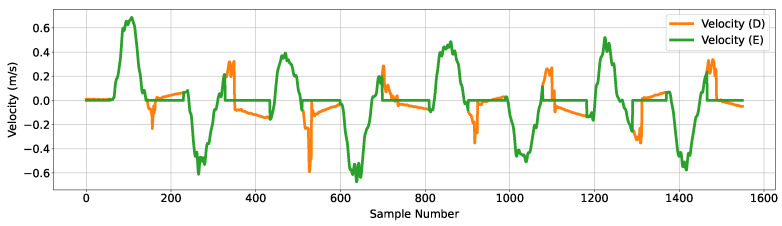
Figure showing velocity data before and after the first stage of processing; the orange trace corresponds to the estimated velocity plot through the first Kalman filter, and the green trace is the processed velocity data with ZUPT.

‘Velocity (D)’ is processed by adopting the ZUPT methodology to remove accumulated errors. In particular, the use of the voltage signal from the non-latching switch along with the ‘State Transition Identification’ process were used to identify periods of zero velocity (‘B’ of Figure 3). Given that the stationary phases have been identified successfully, the estimated velocity values obtained during these phases are explicitly set to zero, hence removing integration errors that have accumulated prior to obtaining estimated position. Additionally, the Kalman filter is reinitialized at the start of the stationary phase to reset the filter state. The results of these changes are shown in Figure 6 as ‘Velocity (E)’, with ZUPT.

**Figure 7:**
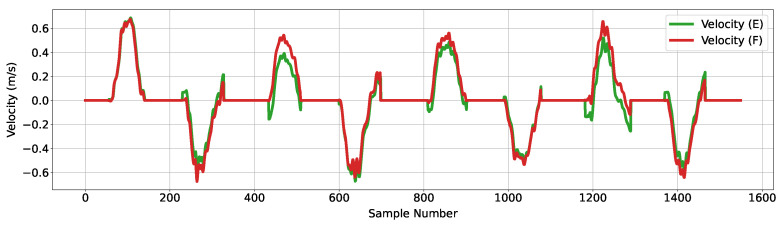
Figure showing velocity data before and after the second stage of processing; the green trace corresponds to the estimated velocity plot immediately after the application of ZUPT, and the red trace corresponds to the velocity data after further error mitigation to remove the intermediary accumulation error that occurs during stationary periods.

The successful identification of stationary periods and the subsequent resetting of the velocity to zero occur when the device has been placed. However, the phases of placement and lift are not instantaneous, and therefore, a stationary drift error component accumulates whilst the device was placed, distorting estimates of subsequent movement phases. To remove this error component, the latest value of the accumulated error in the stationary phase was retained and subtracted from the movement phase data. The outcome of this error subtraction is shown in Figure 7 labeled as ‘Velocity (F)’.

**Figure 8:**
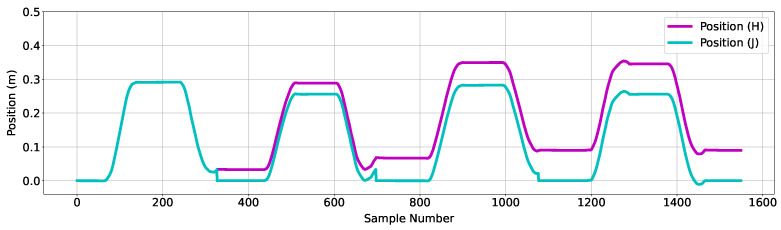
Figure showing associated position data as output of the second Kalman filter used in this algorithm. The purple trace corresponds to position data as output of the second Kalman filter, without integration error mitigation. The blue trace corresponds to the same position data with the inclusion of a function to reset the starting position to zero at every second placement.

The following steps refer to data associated with a second Kalman filter. The ‘Velocity (F)’ shown in Figure 7 is used as an input to a Kalman filter, and the corresponding position output is shown in Figure 8 as ‘Position (H)’. The plot shows the output of a forward reaching movement, over three reaching movements with the average position shown to drift forward by approximately 10 cm. To remove the error for the specific type of movement executed, the system tracks the number of placements completed, with the assumption that the first placement corresponds to the end of the extension phase of movement, and the second placement corresponds to end of the retraction phase of movement. This Kalman filter was re-initialized upon placing the device at the end of the retraction phase, effectively resetting the position estimation process at the end of each completed reaching movement. ‘Position (J)’ in Figure 8 shows the effect of re-initializing the filter on the estimated position.

Although the focus of the methodology is to estimate the position of the hand along the *X*-axis during a forward reach, this algorithm would also be applicable to estimate position along the *Y*-axis (medial/lateral axis).

#### 2.2.3. *Z*-Axis Position Estimation

**Figure 9:**
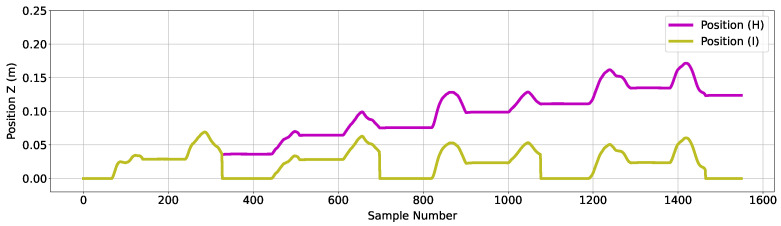
Figure showing associated position data in the *Z*-axis as output of the second Kalman filter used in this algorithm. The purple trace corresponds to position data as output of the second Kalman filter, without integration error mitigation. The olive trace corresponds to the same position data with the inclusion of a function to reset starting position to zero at every second placement.

Figure 9 shows position estimation results for cranial/caudal (*Z*) axis data with the same algorithm steps as described above, labeled as ‘Position (I)’. In a simulated case, the position in this axis returns to zero after every movement. However, displacement error values of approximately 2.5 cm can be observed at the end of the extension phase from the four movements displayed in Figure 9.

**Figure 10:**
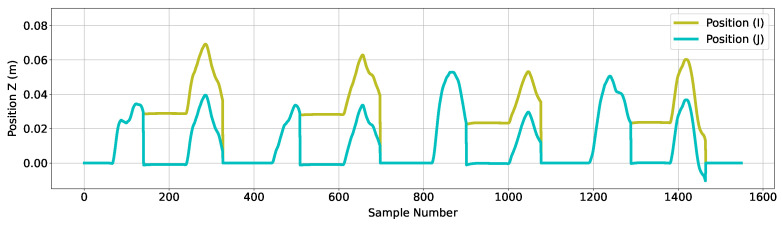
Figure showing the effect of the additional correction mechanism implemented for data associated with the cranial/caudal (*Z*) axis. The olive trace represents the data before the correction mechanism, and the blue trace represents data after the correction mechanism.

Removing this error requires an additional step to the algorithm for the *Z*-axis, and is shown through the inclusion of the ‘Error Mitigation C’ step (shown in Figure 3). Through the known stationary period, this step retains and subtracts the earliest value of the *Z*-axis displacement error from subsequent movement values, effectively resetting the *Z*-axis position after each placement and eliminating the displacement error from movement data. The outcome of this subtraction can be seen in Figure 10 as ‘Position (J)’.

#### 2.2.4. Final Position Output

**Figure 11:**
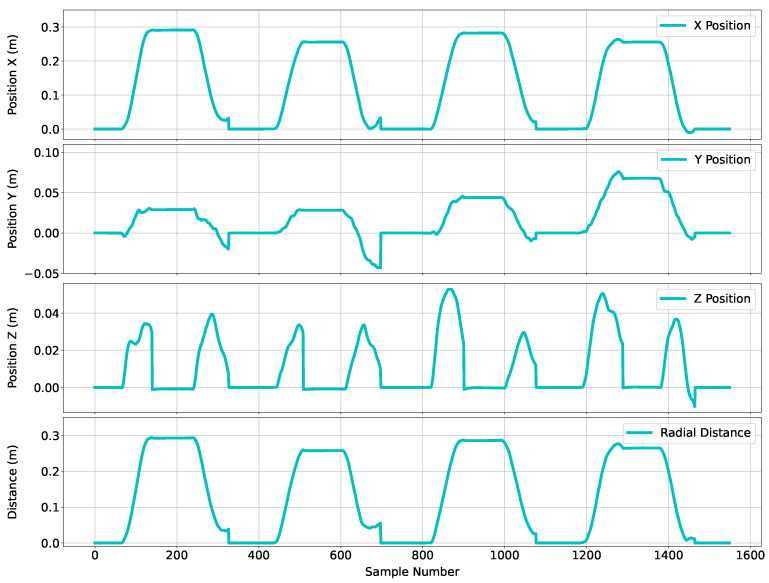
Figure containing four plots corresponding to the estimated position from the Soniccup. The top plot corresponds to the frontal/parietal (*X*) axis, the second plot corresponds to the medial/lateral (*Y*) axis, the third plot corresponds to the cranial/caudal (*Z*) axis, and the bottom corresponds to the radial distance.

Figure 11 revisits the final position estimation plots for all three axes and the radial distance. The radial distance was calculated using Equations (1) and (2).

(1) $$\begin{array}{c} x_{c}=x_{j}-x_{0},\\ y_{c}=y_{j}-y_{0},\\ z_{c}=z_{j}-z_{0}, \end{array}$$

(2) $$r=\sqrt{x_{c}^{2}+y_{c}^{2}+z_{c}^{2}},$$

Equation (1) aligns the origin of data from each system, where $x_{j}$, $y_{j}$, and $z_{j}$ are data associated with the X-, Y-, and Z-axis at sample *j*; $x_{0}$, $y_{0}$, and $z_{0}$ are the first values in the corresponding axis; and $x_{c}$, $y_{c}$, and $z_{c}$ are the corrected data. The radial distance *r* is then computed through Equation (2).

An efficient linear reaching motion (potentially performed by a motorized system) would result in the highest amplitude on the *X*-axis, zero amplitude on the *Y*-axis, and a residual amplitude on the *Z*-axis. Considering that the motion was performed by a human in this case, some amplitude is observed on the *Y*-axis; however, this is not significant for a reaching motion. For the purposes of this study, the *X*-axis output was used for audio feedback, without loss of generality, e.g., the *Y*-axis may also be an input to the sonification system to create audio feedback on lateral motion. The final stage of the process was to translate the motion signals into a form of audio feedback. Details of audio feedback synthesis are described in Appendix A.

## 3. Method

The Soniccup system was designed to produce audio feedback owing to a reaching movement. Although the configuration used to sonify the data is of importance, the data used to create the feedback are a critical factor in generating acceptable audio feedback. A comparison study was carried out to measure the similarity of the position values from the system compared to the associated measurements obtained from a Vicon Nexus system [25] to evaluate whether the position estimation element of the Soniccup is fit-for-purpose. The Vicon system is a gold-standard system for motion capture and is used as ground truth. In line with the majority of the sonification literature [26], a parameter mapping sonification strategy was implemented with the Soniccup. As this strategy linearly converts data into an auditory display, the minima and maxima of the measured signal can be set to an arbitrary minimum and maximum of an auditory dimension. Investigating relative values (as opposed to actual position values) of the data and therefore comparing the similarity of position estimates are of importance in this study.

### 3.1. Procedure

A table and chair were configured in the middle of the Vicon system tracking space. The calibration of both the Soniccup and Vicon systems were completed prior to the study. A volunteer sat facing the Soniccup, positioned on the closest edge of the table. With both systems online, the volunteer performed three sets of 15 reaching movements with the Soniccup in their dominant hand. Each reaching movement consisted of simultaneously raising the system from the table whilst extending their arm to an approximate full reach, before placing the Soniccup back onto the table. The Soniccup was then simultaneously raised whilst the arm was retracted back to the starting position, and then replaced on the table. The volunteer was instructed to perform movements at a normal pace for the first set of data, at a slow pace for the second, and a fast pace for the third.

### 3.2. Data Alignment

Data alignment was achieved through a two-step process. The acceleration data obtained through the Soniccup and the Vicon systems were normalized to a magnitude of positive and negative one. Second, cross-correlation calculations identified the temporal shift value that results in the highest similarity. Equations (3) and (4) capture the process:

(3) $$\begin{array}{c} a_{norm}=a/max\left(a\right),\\ b_{norm}=b/max\left(b\right), \end{array}$$

(4) $$z_{k}=\sum_{n=0}^{||a||-1}a_{n}b_{n-k+(N-1)},$$

where *a* and *b* are the acceleration signals obtained from the Soniccup and Vicon systems, respectively; $a_{norm}$ and $b_{norm}$ are signals after the normalization process; $z_{k}$ is the cross-correlation output at sample difference *k*; *n* equals the sample number; ||a|| is the length of *a*; and *N* is the highest number of samples in *a* or *b*.

The index corresponding to the maximum cross-correlation value was used to identify the difference between the two signals, and was verified through a visual plot. It should be noted that the sampling frequency of both the Soniccup and Vicon systems was 100 Hz, and from visual observation, the two data sets do not expand or shrink sufficiently to warrant the segmentation of data for alignment purposes.

### 3.3. Data Processing

In using the pressure values from the Soniccup as a reference, periods of movement and non-movement were labeled onto data from each motion capture system. Periods of non-movement were removed from the captured data to produce temporally aligned movement data. As the axis alignment of the systems was entirely manual, data obtained from the motion capture systems were converted to radial distance through Equations (1) and (2) (Section 2.2.4). Data were normalized to a maximum of one following conversion through Equation (5):

(5) $$r_{norm}=r/max\left(r\right),$$

where *r* and $r_{norm}$ are subsequently processed through Equation (6) to calculate similarity through a Mean Squared Error (MSE) metric, where *n* is the total number of samples, *d* corresponds to the data used, i.e., *r* or $r_{norm}$, and *i* is the *i*th sample of data.

(6) $$MSE=\frac{1}{n}\sum_{i=0}^{n-1}\left(d_{i}-{\hat{d}}_{i}\right),$$

## 4. Results

Table 1 presents the results of the three sets of movement data at the normal (Movement Set 1), slow (Movement Set 2), and fast (Movement Set 3) paces. The results include the average mean and standard deviation of the duration and peak speed of the performed movements, along with the average mean, standard deviation, and cumulative MSE metrics with and without normalization. The MSE metrics with normalization were used to evaluate the similarity of the data obtained from the systems.

**Figure 12:**
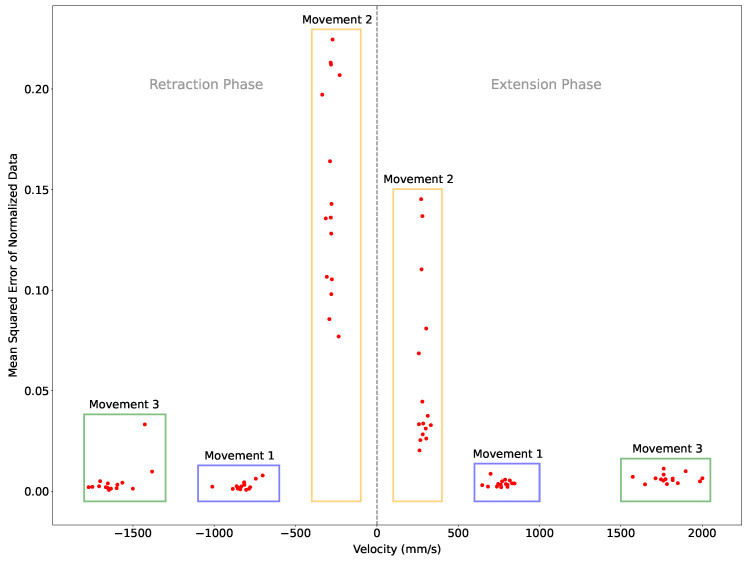
Scatter plot presenting the calculated MSE for each movement. Data points with a positive velocity correspond to the extension phase of the reaching movement, whilst data points with negative velocity correspond to the retraction phase. Boxes enclose plot segments and are labeled with association to the movement set: ‘Movement 1’ for normal speed movement, ‘Movement 2’ for slow speed movement, and ‘Movement 3’ for fast speed movement.

Figure 12 presents the MSE of normalized data as a function of the peak movement velocity from the Soniccup system. Boxes are drawn and labeled to emphasize the associated movement set that the data relates to. The calculated statistical metrics infer high similarity for values between the two systems for Movement Set 1 and Movement Set 3 (Figure 12). For Movement Set 2, however, the MSE metrics indicate that the data are dissimilar, represented by the average mean of the MSE equaling 0.1030, which is two orders of magnitude greater than the next largest average mean of the MSE (0.0057).

**Figure 13:**
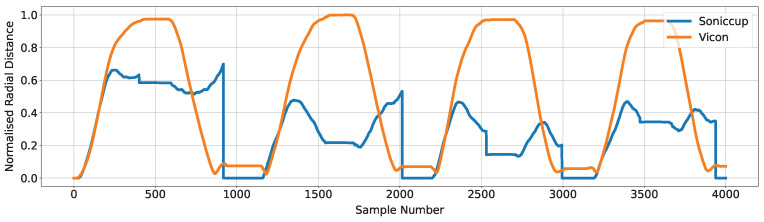
Plot presenting the radial distance obtained through the Soniccup (blue) and Vicon (orange) systems for four movements within Movement Set 2. Data associated with each trace have been normalized so that the maximum data value in the 15 captured reaching movements is equal to one, resulting in the trace associated with the Soniccup showing all data points in the first four reaching movements to be <0.7.

Figure 13 shows two plots of the first four movements captured by each system and provides a visual representation of the performance of the Soniccup system for Movement Set 2. The effect of the normalization process (Equation (5)) on the Soniccup data is clearly evident, as the trace associated with the Soniccup does not reach a value of 0.7; consequently, the value of $r_{max}$ exists on a movement after the first four movements, and the relative values obtained through the system are highly variable between each movement. The MSE for the normalized data values for each movement shown in Figure 13 are 0.0337, 0.1361, 0.1452, and 0.2122, respectively.

## 5. Discussion

The research presented details a position estimation algorithm using ZUPT for upper limb motion tracking. This method was developed with movement sonification in mind; however, this method is not application-restricted to movement sonification. The system—‘Soniccup’—uses the described algorithm to convert gravity-free acceleration values into position estimates, conditioned to enable a display through audio feedback. The process of converting position estimates into an auditory display is described in Appendix A. The research expands the contributions of Bai et al. [21] and Comotti et al. [20] through the creation of a system that enables online upper limb motion tracking for forward reaching movements. Further system development is required prior to its acceptance as a rehabilitation tool, with improvements in slow movement performance a priority.

### 5.1. Comparison Study

A study using similarity metrics was carried out to evaluate the accuracy of the position estimates obtained from the Soniccup system through a comparison of the position data obtained through the Vicon Nexus system with the goal to confirm the validity of the system in sonifying movements performed by people with a range of functional competency, and in turn to provide evidence of the potential suitability of the system for movement sonification-based rehabilitation applications.

The evaluation methodology adopted was founded on one movement performer, through instruction, executing 15 forward reaching movements at a slow, normal, and fast pace, relating to an average mean speed of 283.69 mm/s, 796.17 mm/s, and 1705.35 mm/s, and in assuming that the range of movement was consistent, the average duration of movement was 2.94 s, 0.91 s, and 0.53 s, respectively. As visually presented in Figure 12, the speed of the performed movements was consistent for each instruction. Utilizing the average mean MSE values as a metric, the system produced a positional output of high similarity to the Vicon system for movements of duration less than a second; however, as the movement duration increased, the similarity decreased, manifested through greater MSE values. Typically, upper limb movements performed by stroke survivors are slower and jerkier than non-neurologically affected individuals [27]; these combine to form longer durations of movements, and hence, the results of the slow pace movements in this study are critical for evaluating the applicability of this technology for stroke rehabilitation. A primary reason for decreased performance in the slower movement is the extended time periods between each placement of the Soniccup object, i.e., extended periods of time between the activation of the zero-velocity updates. The results corroborate the findings of Bai et al. [17] that highlight the effectiveness of the technique, reliant on the identification of regular zero-velocity periods. Improving position estimates for longer movement periods through dead reckoning remains a challenge.

### 5.2. Soniccup Latency

The results of the position estimation algorithm shown in this paper rely on the synchronous processing of the data pertaining to the start of movement as obtained from the switch (and therefore, the cessation of ZUPT) and the accelerometer. In the current implementation, this synchronous processing was performed by delaying the accelerometer data to temporally match the switch data. The process of the movement performer lifting the Soniccup, the momentary push-to-make switch changing state from on to off (due to a discrepancy between the total travel distance and the electrical distance), and the State Transition Update step (described in Section 2.2.1) creates a start-of-movement lag for the switch data relative to the accelerometer. To generate the reported results, a manual delay of 210 ms was added to the pipeline for the accelerometer data to ensure synchronicity with the switch signal and therefore assure that all movement values were subjected to integration and none were effectively filtered out as a consequence of ZUPT. As a consequence, the theoretical total time between the initial acceleration measurement and the corresponding position estimate was calculated to be 230 ms. The process to obtain these time values, along with a detailed description of the system delay, is described in Appendix B.1.

Although the system has been proven to provide audio feedback online, it is highly desired that any solution provides real-time audio feedback of movements, enabling synchronous concurrent feedback, which has been touted to improve the learning and retention of new motor skills [28]. The algorithm described in this paper results in a 230 ms delay, which exceeds audio-motor real-time perception [29]. The inclusion of the push-to-make switch is the primary reason for the large system delay.

The push-to-make switch was selected for a number of desirable properties, including low cost, low actuation force, and momentary activation; however, the non-negligible difference of the switch electrical distance with respect to the total distance was a negative factor to system operation. Currently, the Soniccup is setup so that as the object sits flush on a surface, the switch is compressed to the total travel distance. The total travel distance of the switch is 2 mm, whereas the electrical distance, i.e., the distance required to change switch state, is 0.8 mm, creating a 1.2 mm gap between the system being grasped and the switch changing state. One approach for correcting the issue is to engineer the depth of the cavity on the base of the system so that the switch only reaches the electrical distance when the system sits flush on the surface. The adjustment would result in a reduction in the discrepancy at the start of movement and, in turn, decrease the system latency for position estimation. Alternatively, replacing the switch with a smaller difference between the electrical distance and total distance (ideally <0.01 mm) would also suffice.

Another system component that created delay was the need to check for purposeful changes in switch state. Here, a design decision was to use the initial state change sample and eight subsequent samples to identify a purposeful change in switch state, resulting in a delay of 80 ms. Substituting the use of a push-to-make switch with an alternative technology as a movement/non-movement identifier would be an effective solution.

### 5.3. Future Developments

To reiterate, the position estimation method via the Soniccup is a promising method to acquiring position estimates from gravity-free acceleration. The technology is intended to be used by persons with upper limb impairment, such as stroke survivors, for practice in lifting and placing tasks. As such, the results of the slow pace data are of great importance and require great improvement. Evidently, the ZUPT method used to condition velocity values have limitations in effectiveness when the stationary markers are beyond a temporal threshold. Questions remain as to whether the inclusion of alternative sensor data could be included in the method to improve position estimates, particularly given the availability of orientation data. However, given the known high movement variance of compensatory movements performed by stroke survivors [30], the inclusion of orientation data into position estimates may limit the generalizability of the created system.

Alternative improvements can be carried out to reduce the observed latency between movement and output (as described in Section 5.2), and to improve the efficiency of the method. The described implementation contains a Kalman filter on either side of the ZUPT process, which could be combined together to remove redundant implementation. However, these are peripheral improvements to the technology and should be considered once the position estimation performance has improved.

## 6. Conclusions

An online movement sonification system using a single IMU sensor to measure acceleration and derive position estimates for forward reaching movements was designed and evaluated. The results show that the position estimation achieved by the system has high similarity to the measured position obtained through a Vicon system for a restricted set of movements.

Position estimates become strongly dissimilar for longer durations of movement, where slow movements obtained average MSE values of 32,994.65 ${\mathrm{mm}}^{2}$, whereas normal and fast movements obtained average MSE values of 7413.21 ${\mathrm{mm}}^{2}$ and 10,314.38 ${\mathrm{mm}}^{2}$, respectively, and as such, the conversion of estimates for slow movements to the audio domain generates unrelatable audio feedback. Further work would be required to improve the position estimation performance of the system prior to evaluation for slow-movement performers together with further reductions in the latency of the position estimation algorithm to improve the perceived synchronicity of the audio feedback.

## Figures and Tables

**Table 1:** Table of key results obtained from comparison study.

Movement Set	1	2	3
Mean (SD) Movement Duration (s)	0.91 (0.08)	2.94 (0.37)	0.53 (0.04)
Mean (SD) Peak Speed (mm/s)	796.17 (68.01)	283.69 (23.40)	1705.35 (139.26)
Mean (SD) of MSE of Normalized Data	0.0034 (0.0019)	0.1030 (0.0640)	0.0057 (0.0058)
Accumulation of MSE of Normalized Data	0.1005	3.0893	0.1697
Mean (SD) of MSE (${\mathrm{mm}}^{2}$)	7413.21 (2838.81)	32,994.65 (13,644.69)	10,314.38 (5494.36)
Accumulation of MSE (${\mathrm{mm}}^{2}$)	222,396.22	989,839.36	309,431.51

**Figure A1:**
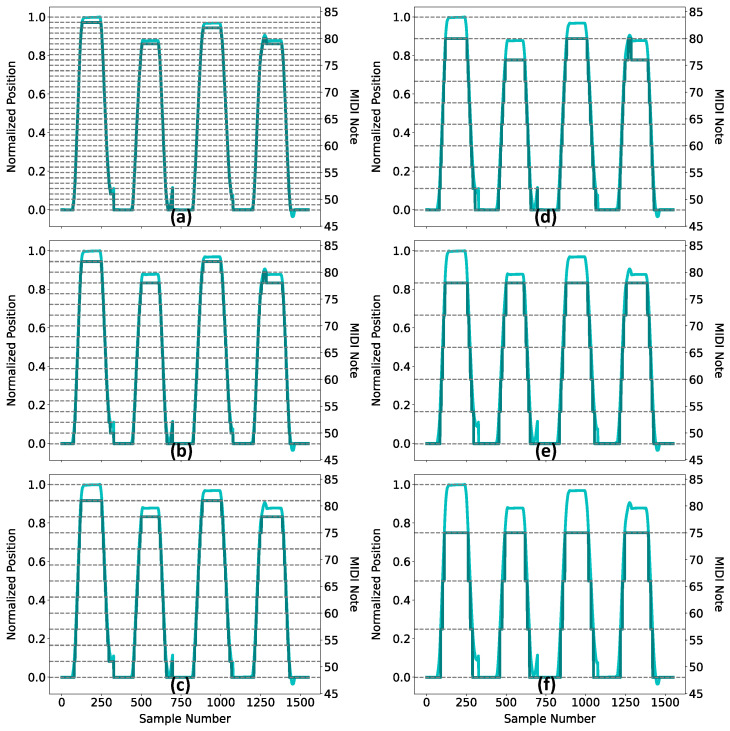
Figure showing six plots corresponding to the effect of altering the audio resolution parameter as stated in Equation (A3) on ‘Movement Set 1’. Labels (**a**–**f**) correspond to the numeric values 1, 2, 3, 4, 6, and 9 used for the $n_{steps}$ parameter. Dark green traces correspond to generated MIDI notes from position estimates shown through light blue traces.

**Figure A2:**
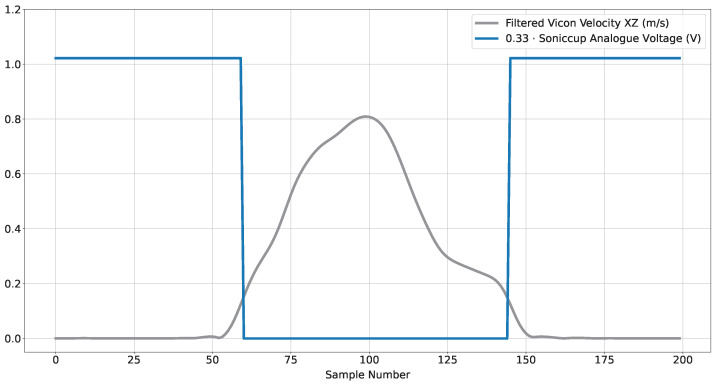
Figure displaying the calculated velocity obtained from the Vicon system, and the analogue voltage recordings corresponding to the switch state obtained through the Soniccup system, with magnitude reduction in this trace by a third.

**Figure A3:**
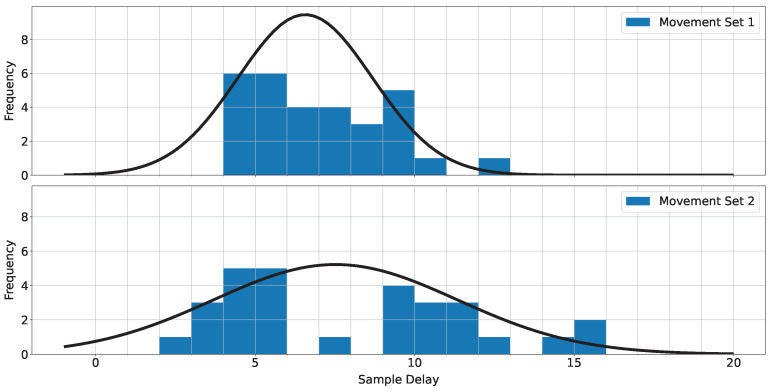
Figure illustrating the variation in the sample offset between the theoretical start of movement and the change in switch state due to the lifting of the Soniccup for ‘Movement Set 1’ (**top**) and ‘Movement Set 2’ (**bottom**). A density plot is shown in both plots as a black trace. The positive offset indicates that the movement began before the switch changed state.

**Figure A4:**
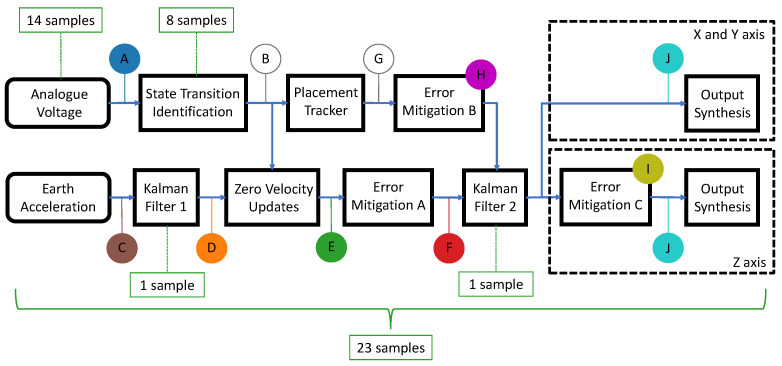
Flowchart presenting sources of delay associated with the start of movement that accumulate during the Soniccup methodology. This figure shows Figure 3 with added annotations corresponding to sources of delay. The total delay is shown as 23 samples, with one sample’s worth of delay equaling 10 ms.

## Data Availability

The raw data supporting the conclusions of this article will be made available by the authors upon request.

## Appendix A. Soniccup Sonification

Translating motion signals into audio feedback is the final stage of the movement sonification process. For the Soniccup system, position estimates were chosen to generate audio feedback. Position estimates are sonified through a parameter mapping process that linearly maps data values to audio pitch, viz. as the movement performer extends their arm, the audio pitch rises, and when the movement performer retracts their arm, the opposite occurs.

Audio output can be presented through ‘Musical Instrument Digital Interface (MIDI) notes’ [31]. MIDI is a communication standard for digital musical instruments and related devices for playing, editing, and recording music. MIDI note values range from 0 to 128, which is wider than the grand piano, of which the lowest note (A0) corresponds to MIDI note 21, and the highest note (C8) corresponds to MIDI note 108. Notes at the high end of the spectrum generate high treble, which may create discomfort to the user, while notes at the low end of the spectrum are harder to perceive. Therefore, the audio was designed with the lowest note at MIDI note 48 (C3) and the highest at MIDI note 84 (C6), limiting the range to three octaves.

The MIDI note mapping was created using Equations (A1)–(A3). Initially, the note range $note_{min}$ to $note_{max}$ is scaled over the position range $p_{min}$ to $p_{max}$ to obtain the scaling factor *r*:

(A1) $$r=\frac{\left(note_{max}-note_{min}\right)}{\left(p_{max}-p_{min}\right)},$$

where $note_{min}$ = 48 and $note_{max}$ = 84, corresponding to the upper and lower boundary of MIDI notes available as output; and $p_{min}$ = 0 and $p_{max}$ = maximum position, corresponding to the start and end values of the reaching motion. Specifically, $p_{max}$ is a value estimated based on the range of movements captured previously through the Soniccup; therefore, trial movements are required prior to online sonification. The scaling factor *r* is calculated at the system initialization, and hence requires position estimates for the start and end of movement.

With the aid of the *r* scaling factor, the audible note $n_{i}$ at position $p_{i}$ can be obtained using the equation

(A2) $$n_{i}=\left\lfloor r\cdot p_{i}\right\rfloor +note_{min},$$

Note that the product $r\cdot p_{i}$ is floored to quantize the note output, as decimal notes are not meaningful. This will result in all 36 notes in the range $note_{min}-note_{max}$ to be audible, and small deviations in $p_{i}$ will result in fluctuating audio output. To avoid noisy audio output, note quantization can be made coarser by increasing the number of steps ($n_{steps}$) between audible note changes, therefore controlling the audio note resolution. To achieve this, Equation (A2) was modified to

(A3) $$n_{i}=n_{steps}\cdot \left\lfloor \frac{r\cdot p_{i}}{n_{steps}}\right\rfloor +note_{min},$$

Figure A1 displays multiple plots showing the effect of the $n_{steps}$ parameter on the conversion process from normalized position estimates to MIDI notes. The dotted horizontal lines represent the cut-off threshold for each audio note where $n_{steps}$ equals 1, 2, 3, 4, 6, and 9, which correspond to plots (a), (b), (c), (d), (e), and (f). As the resolution parameter increases, the number of audio notes displayed decreases from 36 to 5, resulting in an increase in pitch interval for each note transition and an increase in the travel distance required per note change.

Note that the aim of the movement sonification system is to capture the relative motion patterns and generate similar audio feedback for similar patterns; therefore, position estimates for the system are normalized using max normalization and result in values in the range [0,1] using the equation

(A4) $$p_{norm}=p_{i}/p_{max},$$

where $p_{norm}$ is the normalized position value.

For the Soniccup system, the $n_{steps}$ parameter was set to three, resulting in 13 audible MIDI notes. However, through experimentation, it was observed that at the near maximum extremity of the reach, the audible output created trills (rapid alternations between two notes). The trills were a consequence of the hand hovering around a threshold between MIDI note changes when the arm extension reached 100% and resulted in the movement performer overextending to mitigate the trill. To mitigate the trill through the sonification configuration, the position estimate was updated to saturate at 95% of the maximum position. This was implemented through Equation (A5).

(A5) $$p_{i}=min\left(p_{i},0.95\cdot p_{max}\right),$$

The saturation on the high end helps to discourage overextension of the movement performer, which in turn promotes a healthy reaching goal for users of the system. Consequently, this saturation removes the last note of the MIDI range, and therefore, this configuration displays 12 audible MIDI notes.

Likewise, saturation was set for the lower extremity of reach, viz. when the arm retracts beyond the starting position and the position estimates becomes negative, instead of the audio output emitting notes below the selected MIDI range, the lowest audible note $note_{min}$ is maintained, as implemented using Equation (A6):

(A6) $$p_{i}=max\left(p_{i},p_{min}\right)$$

which was set to 48. Through Equations (A5) and (A6), a sonification range was created, where movement captured outside of designated area create saturation to sustain a constant audio note ranging from (and including) 48 to 81 for the low and high ends, respectively.

## Appendix B. System Latency

The Soniccup is an online movement sonification system that provides audio feedback based on movement kinematics relating to the tracked hand of the movement performer. In Section 2.2.1, the switch transition identification used to identify the Soniccup placement and lift resulted in a delay of 80 ms for the position estimation. However, using the methodology as described in Section 2.2 results in other sources of delay, which are described in this section.

### Appendix B.1. Aligning Start of Movement

To estimate position effectively, the sensor streams corresponding to the acceleration and switch state must be temporally aligned at the start of movement. Failure to achieve this produces position estimates through the Soniccup methodology that do not represent the movements performed. Figure A2 shows an example of an extension movement that has been captured through the Soniccup and the Vicon system in parallel.

Data from the two systems have been temporally aligned using the same process as described in Section 3.2. The gray trace in this figure shows the magnitude of the hand velocity (*X*- and *Z*-axis) obtained via the Vicon system, which was low-pass filtered at 6 Hz in both directions [32] to create a zero phase output. The blue trace in this figure shows the switch state prior to the filtering process as described in Section 2.2.1, which has been amplitude-scaled by a third for illustrative purposes. As can be observed in the figure, at the start of movement, the switch transitions from high to low values later than the initial rise in velocity values. This observation is consistent with all movements captured through this system. Addressing this start-of-movement temporal misalignment is a necessity for the Soniccup methodology.

To remove the effects of temporal misalignment requires the delaying of a signal so that the start of movement at both data streams is synchronized. To achieve this for the start of movement, the acceleration data stream has to be delayed by $X_{i}$ samples to match the switch state signal. However, the value of $X_{i}$ varies for different reaching movements. Understanding the magnitude and range of values of $X_{i}$ partly permits the evaluation of the Soniccup.

To obtain an estimate of $X_{i}$, all reaching movement data obtained via the method in Section 3 for ‘Movement Set 1’ and ‘Movement Set 2’ were analyzed to identify the start of movement from the Vicon system (velocity magnitude of *X*- and *Z*-axis) and Soniccup system (switch state). ‘Movement Set 2’ represents reaching movements performed at a slow pace and was included in this estimation process as it was expected that the slower movement resulted in larger temporal misalignment between the two signals, compared to ‘Movement Set 1’. For data associated with the Vicon system, the first value above a velocity threshold of 0.02 m/s [32] was used to determine the start of movement, and for data associated with the Soniccup, the first value below a threshold of 0.1 V was used. The extracted sample numbers were then compared. This process resulted in a total of 30 data points for $X_{i}$ in ‘Movement Set 1’ and 30 data points in ‘Movement Set 2’. Of the data points obtained in each set of data, statistical outliers were identified using Equations (A7) and (A8),

(A7) LO=Q1−(1.5·IQR),

(A8) HO=Q3+(1.5·IQR),

which identified one outlier in ‘Movement Set 2’ that was excluded from the analysis. No outliers were identified for ‘Movement Set 1’. The resulting data for $X_{i}$ are displayed through Figure A3. Figure A3 shows the measured variable $X_{i}$ for each movement within ‘Movement Set 1’ (top) and ‘Movement Set 2’ (bottom), along with a density estimate curve for each. The means and standard deviations for each density estimate curves are shown in Table A1.

**Table A1:** Table showing the means and standard deviations for the density estimate curves shown in Figure A3.

Movement Set	Mean	Standard Deviation
1	6.567	2.108
2	7.517	3.820

In the current implementation of the Soniccup, a design choice was made to ensure that the system latency is constant. To set a constant system delay for the Soniccup, $X_{i}$ must also be modeled as a constant ($X_{const}$). Setting $X_{const}$ to be a value smaller than the actual delay would result in the zeroing of movement data at the early stages of movement and needs to be avoided for the position estimates to effectively represent the performed movements. Setting $X_{const}$ to be a value larger than the actual delay results in the inclusion of data prior to the start of movement in the synthesis of position estimation. Given that the ZUPT methodology explicitly zeros these values, the inclusion of these extra values does not affect the position estimates, and therefore, overestimating $X_{const}$ is preferable to underestimating. The compromise, however, is that a larger $X_{const}$ value leads to a larger system latency. Assuming that these values form a normal distribution, a value of $X_{const}$ corresponding to 95% of the data values can be calculated using the mean and standard deviation of an attained density estimation curve. For this analysis, the measurements from ‘Movement Set 2’ were used to obtain a conservative estimate of $X_{const}$ via Equation (A9):

(A9) $$X_{const}=\mu +Z\sigma ,$$

where μ is the mean value, σ is the standard deviation unit value, and *Z* is the Z-score corresponding to a level of confidence of 95%. From this equation, $X_{const}$ = 15.004.

$X_{const}$ was calculated from the data obtained comparing the sample numbers corresponding to the start of movement from the switch state data obtained through the Soniccup system and the *velocity* data obtained via the Vicon system. To convert the calculated measures to compare the switch state and acceleration data streams associated with the Soniccup (*X*), the calculated mean value and therefore the calculated sample discrepancy value were reduced by one for the start of movement. Equation (A10) shows the transition from $X_{const}$ to *X*:

(A10) $$X=\lfloor X_{const}-1\rfloor ,$$

where the intermediary value is rounded down to the nearest integer. Therefore, *X*, as calculated through a combination of Equations (A9) and (A10), is 14 samples, and therefore, the acceleration signal would need to be delayed by 140 ms.

### Appendix B.2. Algorithm Latency

The described latency is the time between the motion capture measurement and the synthesis of the output position. The delay as created by factors external to the position estimation methodology is considered to be less than 1 ms and, therefore, has not been included. Figure A4 shows a model of the accumulated delay through the stages of the Soniccup position estimation methodology, where one sample corresponds to a delay of 10 ms.

Sources of delay include the filtering mechanism used to remove momentary changes in state, resulting in a delay of eight samples, and the integration mechanism performed through the two Kalman filters, which induces a delay of one sample each. To obtain results that represent the movements performed, an additional delay of 14 samples is included into the model, as described in Appendix B.1, to align the input data streams at the start of movement. Subsequently, to temporally align the signals for the ZUPT methodology, a delay mechanism for the acceleration data stream of 21 samples is included in the model.

The total latency of this system is an accumulation of the following three sources, as shown through Figure A4:

- A total of ‘14 samples’ shown above the analogue voltage;
- A total of ‘8 samples’ shown above the State Transition Identification;
- A total of ‘1 sample’ shown below Kalman Filter 2.

This results in a total of ‘23 samples’, as shown in the bottom annotation of the figure. As such, for the current implementation of the Soniccup, from receiving acceleration measurements to obtaining position estimates, the delay time was calculated to be 230 ms.
